# Muscle fat infiltration but not muscle cross-sectional area is independently associated with bone mineral density at the lumbar spine

**DOI:** 10.1259/bjr.20210371

**Published:** 2022-05-05

**Authors:** Qian Yang, Dong Yan, Ling Wang, Kai Li, Wei Liang, Wei Zhang, Yan Dong Liu, Xiao Min Li, Glen M Blake, Natalie Konerth, Xiaoguang Cheng, Wei Tian, Karen Hind

**Affiliations:** Department of Radiology, Tongji Hospital, Tongji Medical College, Huazhong University of Science and Technology, Wuhan, Hubei, China; Department of Radiology, Beijing Jishuitan Hospital, Beijing, China; Department of Radiology, Beijing Jishuitan Hospital, Beijing, China; Department of Radiology, Beijing Jishuitan Hospital, Beijing, China; Department of Radiology, Beijing Jishuitan Hospital, Beijing, China; Department of Radiology, Beijing Jishuitan Hospital, Beijing, China; Department of Radiology, Beijing Jishuitan Hospital, Beijing, China; Department of Radiology, Tongji Hospital, Tongji Medical College, Huazhong University of Science and Technology, Wuhan, Hubei, China; School of Biomedical Engineering and Imaging Sciences, Kings College London, St Thomas’ Hospital, London, United Kingdom; Department of Sport and Exercise Sciences, Durham University, Durham, United Kingdom; Department of Radiology, Beijing Jishuitan Hospital, Beijing, China; Department of Spine Surgery, Beijing Jishuitan Hospital, Beijing, China; Department of Sport and Exercise Sciences, Durham University, Durham, United Kingdom

## Abstract

**Objective:**

Although sarcopenia and osteoporosis are inter-related conditions that are common with advancing age, few studies have explored relationships between muscle quality and bone mineral density (BMD). We investigated age- and sex-specific paraspinal muscle fat infiltration (MFI), muscle cross-sectional area (CSA), and spine volumetric BMD (vBMD) in healthy Chinese adults.

**Methods:**

605 healthy adults aged 20–59 years (340 women, mean age 39.2 years; 265 men, mean age 38.8 years) had axial *T*_2_WI MRI imaging of the lumbar spine and CSA (cm^2^) and MFI (%) were measured in the psoas and multifidus and erector spinae (MF-ES) muscles (L3–L4). MFI measurements were calibrated against a region of interest in an adjacent area of subcutaneous pure fat. L2–L4 vBMD was measured by quantitative CT. Age- and sex-specific subgroups were compared using the Mann–Whitney test. Multiple regression was used to test independent associations of MFI and CSA with vBMD.

**Results:**

Females had lower CSA and higher MFI than males in both the psoas and MF-ES muscles (*p* < 0.001). In females and males, MF-ES MFI increased with age (*p* < 0.001) and in females age-related increases were observed for the psoas muscles (*p* < 0.05). Greater fat infiltration of the MS-ES muscle unit was associated with lower vBMD in both sexes (*p* < 0.001) but not with CSA. Following adjustment for demographic variables and CSA, MS-ES MFI remained predictive of vBMD (β = −0.408 to −0.157, *p* < 0.001).

**Conclusion:**

We have demonstrated that, independent of CSA and demographic variables, MFI of the MF-ES muscles is predictive of lower lumbar spine vBMD in both sexes.

**Advances in knowledge:**

This is the first study to demonstrate that, independent of muscle size and demographic variables, MFI of the paraspinal MF-ES muscles is predictive of lower lumbar spine vBMD in both sexes.

## Introduction

Muscle and bone are considered a functional unit with synchronicity and interactions at the mechanical and biological level.^1^ A loss of integrity in both tissues, leading to sarcopenia and osteoporosis, is common with advancing age and brings substantial health burdens.^2–4^ Sarcopenia, which is defined as a loss of skeletal muscle mass, strength and quality, leads to a decline in physical performance and to frailty in older adults,^5^ leading to disability, low quality of life and mortality.^6^ Muscle atrophy, intramuscular fat accumulation and loss of strength have contributing causes beyond aging that are associated with bone loss, including declining health, inactivity and metabolic or musculoskeletal diseases.^7,8^ Furthermore, although sarcopenia is associated with aging, recent studies have led to a recognition that loss of muscle quality is a process that begins earlier in life.^8,9^

The relationship between sarcopenia and osteoporosis is complex and has not been fully studied. Most studies have investigated the co-existence of the two conditions in older female populations and in association with hip fracture.^10–14^ By comparison, few studies have explored the inter-relationships of muscle and bone in both sexes in populations aged <60 years and knowledge about the early trajectory of the degrading of both tissues is limited.

Skeletal muscle quality and early stage sarcopenia can be accurately assessed using MRI,^4^ which has high spatial resolution and excellent soft tissue contrast for detecting changes in muscle composition while avoiding radiation exposure.^14^ As such, MRI is an ideal method for both research and for clinical use in community screening.

The spine, which is predominantly comprised of trabecular bone, is a common skeletal site for bone loss in both sexes, and the prevalence of vertebral fracture in populations aged over 60 years ranges from 9 to 26%.^15^ Several studies have investigated age-associations between paraspinal muscle cross-sectional area (CSA) and muscle fat infiltration (MFI) in healthy adults and a recent study reported a correlation between MFI and lumbar spine vBMD in a small Chinese population.^16^ However, no study has yet explored both CSA and MFI in relation to vBMD with adequate statistical power to investigate both sexes independently. In addition, there is a need for population-specific reference ranges given reported differences by ethnicity.^14,17–20^

The aim of this study was to investigate associations between lumbar paraspinal muscle properties and lumbar spine vBMD in adults aged <60 years. A secondary aim was to provide age and sex reference data for lumbar paraspinal muscle properties using conventional *T*_2_ weighted MRI images in healthy Chinese adults that may be useful for future studies in this field.

## Methods and materials

### Study participants

605 healthy adults were recruited between December 2013 and February 2016 from communities within the vicinity of the Beijing Jishuitan hospital. The subjects included in the study were participants in an ongoing research study on degeneration of the spine and knee. Details of this cohort were reported previously.^21^ The volunteers were widely distributed in terms of age between the third and sixth decade. Inclusion criteria were healthy adults up to the age of 60 years who were able to provide informed consent. Exclusion criteria were pregnancy, metal implants, lumbar spine fractures, lumbar surgery history, other serious comorbidities such as infections, tumors, diabetes mellitus, neurological diseases and muscle disorders, or claustrophobia. The local research ethics committee approved the study and all participants gave written informed consent. Data on age, height, weight, waist circumference and hip circumference were collected and body mass index (BMI) was calculated as the weight in kilograms divided by the squared height in meters.

### MRI protocol and scan acquisition

MRI scans were obtained using a 3 T unit (Philips Healthcare, Best, The Netherlands). Each participant had a routine lumbar spine scan and axial *T*_2_ weighted images (TR/TE, 3391/120) obtained at the L3–L5 intervertebral disk levels. The field of view of the axial MR images was 160 × 178 mm, and the slice thickness was 3.5 mm, with slice gaps of 0.4 mm. Matrix sizes were 248 × 198 mm, with a voxel size of 0.85 × 0.87 mm and flip angle 90°. Images were stored in DICOM format for processing.

Quantitative measurements of muscle were performed on the MRI *T*_2_WI axial images using OsiriX (v. 5.8.5, Pixmeo, Geneva) software.^22^ Each muscle region of interest (ROI) from *T*_2_ weighted axial images taken from a single slice was determined by using the OsiriX pencil tool and was manually traced using an external mouse. T2 axial images have been used previously to evaluate paraspinal muscle morphology and composition.^23^ Measurements were obtained from a single slice at the upper border of each disc at the level of the superior endplate of L3/L4.

**Figure 1.**
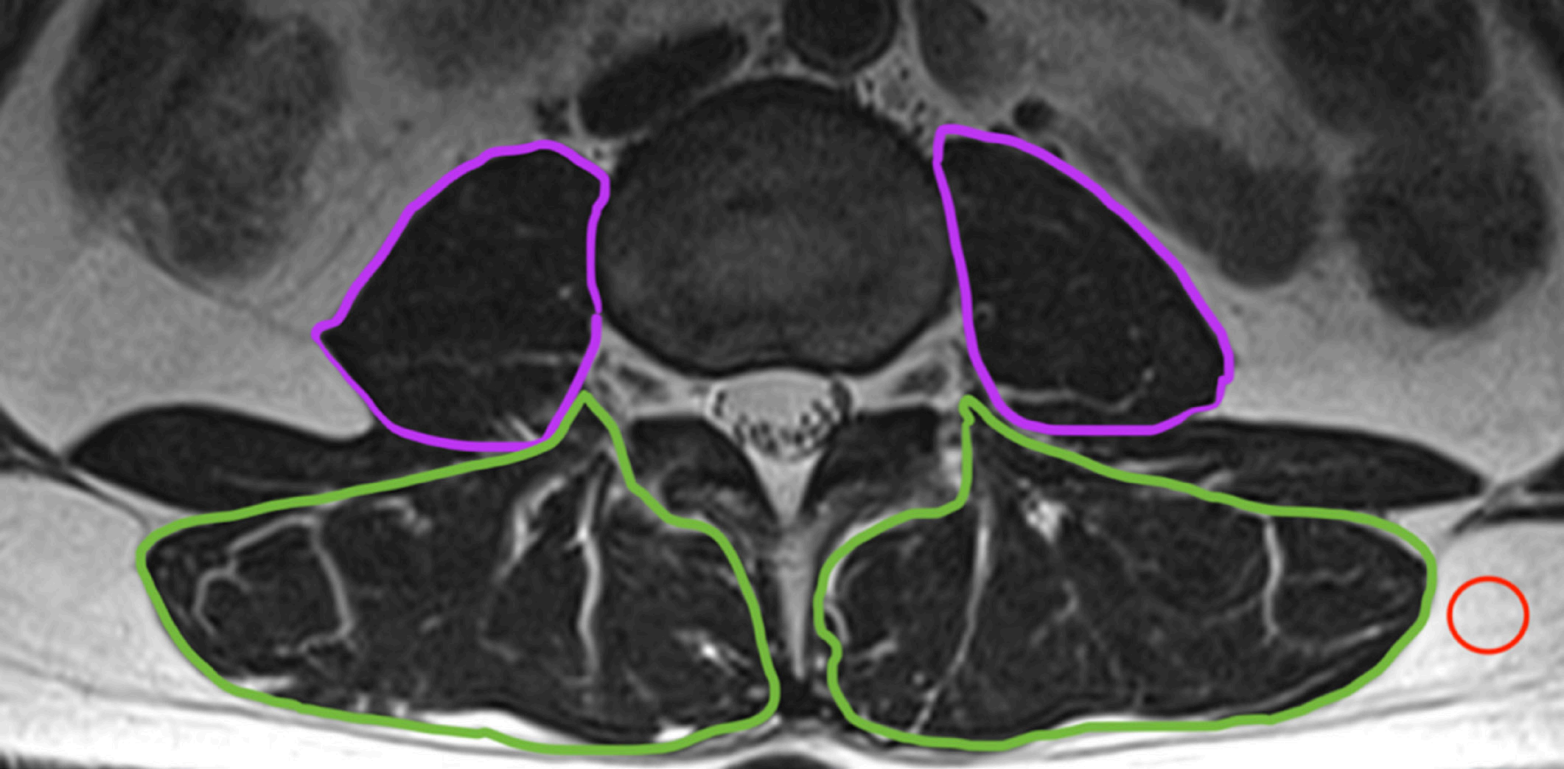
*T*_2_ weighted MRI showing segmentation for CSA and MFI analysis. The violet line represents the measurement of psoas muscle obtained using OsiriX software, while the green line represents the measurement of MF and ES muscles as a unit bilaterally and the red circle represents the measurement of a pure fat ROI. CSA, cross-sectional area; ES, erector spinae; MF, multifidus; MFI, muscle fat index; ROI, region of interest.

We defined ROIs on the right and left sides for the multifidus and the erector spinae as a single unit and similar ROIs for the psoas muscle (Figure 1). CSA was measured by manually constructing polygon points around the outer margins of the individual muscles excluding the outer muscular fat. We chose segmentation of the multifidus and erector spinae muscle as a unit based on visible muscle boundaries, which did not include epimuscular fat, based on the report of Berry et al analyzing the reliability of methods for defining ROIs in lumbar paraspinal muscle.^24^ Additionally, a ROI with an area of 1.4 cm^2^ representing pure fat was placed in subcutaneous adipose tissue adjacent to the spine and the measurements used to calculate the MFI index to assess the extent of fat infiltration in paraspinal muscle based on the method described by Elliott.^25^ The measurements obtained in the 605 study subjects were total transverse sectional CSA in the muscle ROIs and MFI, which was calculated by dividing the mean signal of the total muscle ROI by the subcutaneous adipose tissue ROI signal. Due to MR characteristics, signals from muscle and fat in any ROI can vary over a large range in different subjects. However, the ratio between the muscle and fat signal is relatively stable.

### QCT protocol and acquisition

The lumbar vertebrae from L2–L4 were scanned with a CT scanner (Aquilion PRIME ESX-302A, Toshiba Medical Systems Corporation, Otawara, Japan). A calibration phantom (Mindways Inc., Austin, TX) was placed beneath the spine and scanned simultaneously according to a standard protocol.^21^ The scan parameters were as follows: 120 kV, 187 mAs, field of view 40 cm, slice thickness 1 mm, and reconstruction matrix 512 × 512. After scanning, the CT data sets were transferred to a workstation for further analysis with the Mindways QCT Pro software (v. 5.0.3). The ROIs were defined as oval-shaped areas containing the largest area of trabecular bone, not including cortical bone or the basivertebral plexus. For calibration a European Spine Phantom (ESP-145) (QRM GmbH, Möhrendorf, Germany) was scanned 10 times. Raw vBMD measurements produced by QCT Pro software were adjusted to the manufacturer-calibrated values for the ESP-145 phantom using a linear regression fit to the three ESP vertebrae. The mean vBMD of the three vertebrae was used for statistical analysis.

### Statistical analysis

The statistical analysis was performed using SPSS Statistics v. 25.0 (IBM Corp., Armonk, NY). The participants were divided into four age groups, 20–29, 30–39, 40–49 and 50–59 years. The highest age group was 50–59 years in males and 50–58 years in females. Initial analyzes described the participants’ characteristics according to age and gender. The Shapiro–Wilk test was used to evaluate normality and normally distributed variables expressed as means ± SDs and non-normal variables as medians and interquartile ranges. Differences in CSA, MFI and vBMD between age groups and sexes were evaluated using the Mann–Whitney test. Correlations of muscle measurements with demographic characteristics were investigated using Spearman’s correlation coefficient. A correlation coefficient <0.1 was considered negligible, between 0.1–0.3 weak, 0.3–0.5 moderate and 0.5–1.0 strong. To investigate the independence of associations, skewed variables (all variables except age, height and vBMD) were log transformed and multiple regression was performed adjusting for CSA and demographic variables (age, height, weight, and waist circumference). *p* < 0.05 was considered significant.

Intraobserver precision was evaluated from measurements of 30 randomly selected MR images performed twice by a single radiologist with time interval >3 months. Interobserver precision was evaluated from measurements of another 30 randomly selected MR images that were analyzed independently by two radiologists. Intra- and inter-rater reproducibility was determined using the intra- and interclass correlation coefficients (ICCs). ICC values for the intra- and interobserver reproducibility for the CSA and MFI MRI measurements ranged from 0.938 to 0.992 and 0.854 to 0.988 respectively, indicating reliable measurement methods.

## Results

**Figure 2.**
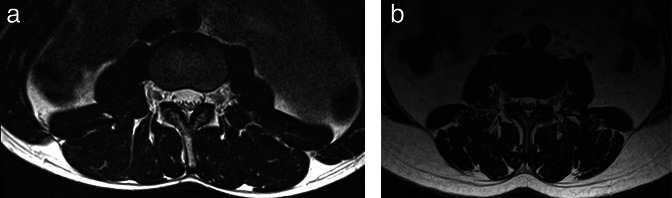
*T*_2_ weighted magnetic resonance images of participants with lower and higher MFI values in the MF-ES muscles respectively. (**A**) 31-year-old female with an MFI of 15.6%; (**B**) 51-year-old female with an MFI of 35.7%. ES, erector spinae; MF, multifidus; MFI, muscle fat index.

Demographic statistics of the participants are shown in Table 1. Data for CSA, MFI and vBMD by age and sex are presented in Table 2. Figure 2 shows *T*_2_ weighted MRI images of two participants with lower and higher MFI values in the MF-ES muscle respectively. No significant differences in CSA or MFI were found between the right and left sides and therefore the mean was used to explore differences between age groups.

**Table 1.** Basic demographics for males and females [mean and (SD)]

	Males	Females	*p*-value (sex)
Age (y)	38.8 (8.1)	39.2 (8.4)	0.516
Height (cm)	172.1 (5.9)	160.6 (5.6)	<0.001
Weight (kg)	78.2 (12.1)	61.5 (10.3)	<0.001
BMI (kg/m^2^)	26.4 (3.7)	23.9 (3.8)	<0.001
Waist circumference (cm)	91.1 (9.5)	79.7 (10.8)	<0.001
Hip circumference (cm)	100.8 (6.4)	96.1 (7.0)	<0.001

BMI, body mass index; SD, standard deviation.

**Table 2.** Distribution of CSA (cm^2^) and MFI (%) of L3/L4 paraspinal muscles and L2–4 vBMD (mg/cm^3^) by sex and age group [median and (interquartile range)]

Age group (N)	MR-muscle CSA (cm^2^)		MR-MFI (%)		QCT-L2-4 vBMD
Male	Psoas	MF-ES unit	Psoas	MF-ES unit	mg/cm^3^
20–29(32)	14.1 (12.2–15.4)	28.2 (25.7–31.0)	12.4 (10.0–16.4)	20.0 (16.6–25.3)	158 (144–171)
30–39 (110)	13.5 (12.2–15.4)	28.2 (25.6–31.4)	12.9 (10.8–16.1)	19.9 (17.5–23.2)	159 (138–180)
40–49 (93)	13.6 (12.2–15.6)	28.5 (25.5–31.4)	13.8 (12.1–17.0)	23.9 (20.3–27.5)	136 (119–153)
50–59(30)	12.6 (10.7–14.0)	26.9 (23.4–30.0)	14.2 (12.2–16.7)	26.2 (23.6–33.0)	118 (102–140)
Total (265)	13.5 (12.1–15.4)	28.1 (25.4–31.1)	13.3 (11.2–16.8)	22.3 (18.7–26.1)	145 (127–166)
**Female**					
20–29 (53)	8.4 (7.6–9.4)	19.5 (18.0–21.5)	16.5 (13.3–18.9)	23.7 (19.9–27.6)	178 (165–201)
30–39 (116)	8.7 (7.2–9.9)	20.1 (18.5–22.5)	15.8 (13.0–18.9)	24.1 (21.3–27.9)	174 (158–200)
40–49 (127)	8.4 (7.4–9.6)	20.4 (18.0–21.9)	16.6 (13.4–19.4)	27.3 (25.0–31.2)	159 (138–182)
50–58(44)	8.2 (7.2–9.1)	20.2 (18.4–22.4)	18.8 (15.6–22.1)	35.8 (30.7–39.6)	119 (100–149)
Total (340)	8.4 (7.3–9.6)	20.2 (18.2–22.1)	16.6 (13.5–19.5)	26.4 (22.8–31.0)	167 (143–187)

CSA, cross sectional area; MF-ES, Multifidus and erector spinae as a unit; MFI, muscle fat index; vBMD, volumetric bone mineral density.

### Age and sex-specific data

There were more females (*n* = 340) than males (*n* = 265). Mean (SD) patient age was 39.0 (8.3) years with no significant difference between males and females (*p* = 0.52) (Table 1). Other demographic statistics were significantly higher in males. CSA for the psoas and MF-ES unit were higher in males than females in all age groups (*p* < 0.001), whereas MFI for the psoas and MF-ES unit were higher in females (overall: *p* < 0.001; 20–29 years group MF-ES MFI: *p* < 0.05) (Table 2). vBMD was higher in females than males in the three youngest age groups (*p* < 0.001; 50–59 years group: *p* = 0.50).

**Figure 3.**
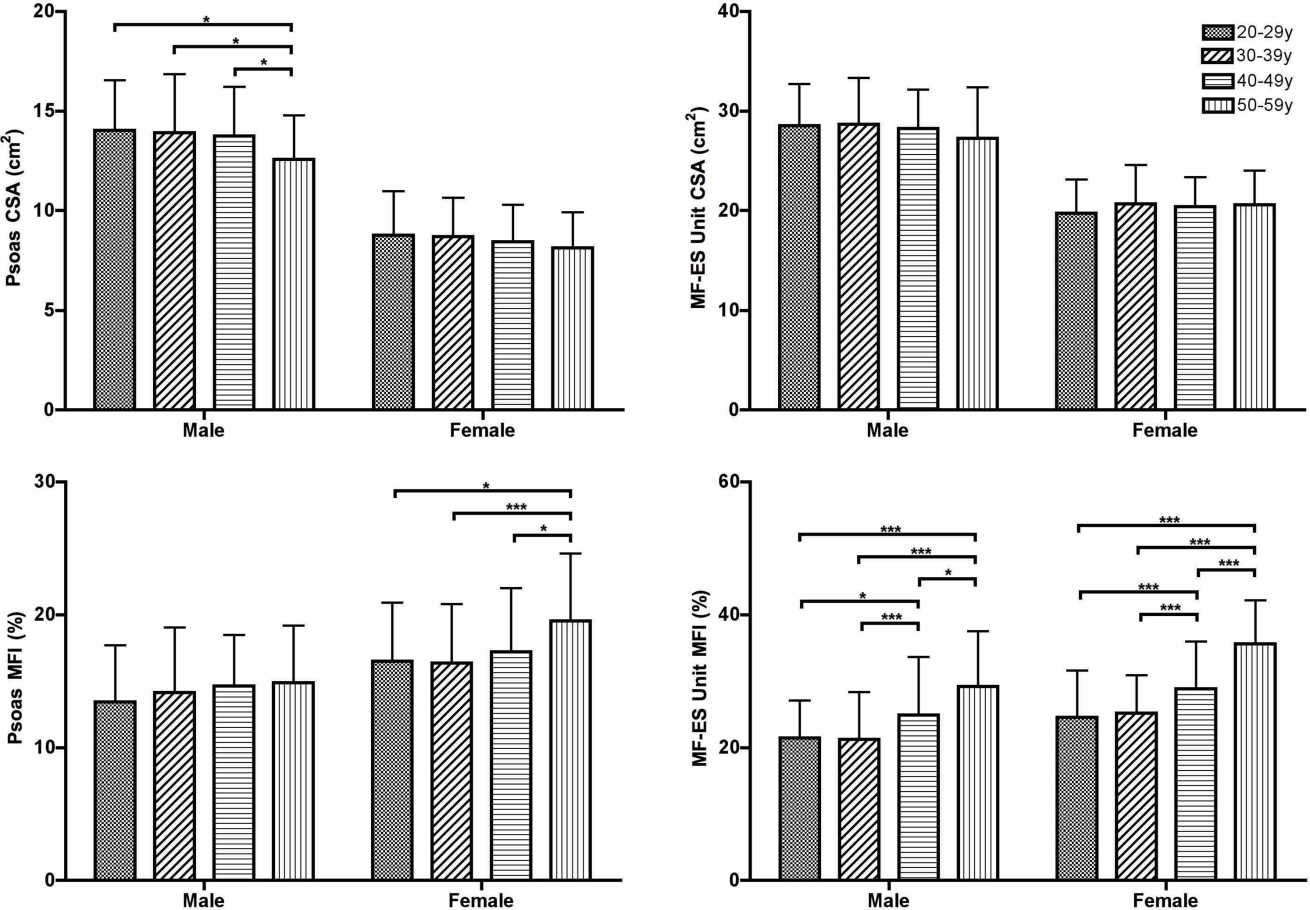
Age-group-averaged MFI (%) and CSA (cm^2^) for both sexes. Multifidus and erector spinae as a unit and psoas MFI and muscle CSA are given respectively. Significant differences of the means between each age groups are indicated by an asterisk (*p* < 0.05) and triple asterisks (*p* < 0.001). CSA, cross-sectional area; MFI, muscle fat index

In females, there was no significant difference in psoas CSA or MF-ES unit CSA between any age group (Figure 3). Both psoas MFI and MF-ES MFI were higher in females in the 50–59 years group than in any of the three younger age groups (*p* < 0.01 and *p* < 0.001 respectively). MF-ES unit MFI was also significantly higher in the 40–49 years group compared with the two younger groups (*p* < 0.001).

In males, psoas CSA was lower in the 50–59 years group compared to the three younger groups (*p* < 0.05), while there was no significant difference in MF-ES unit CSA between any age group. Although there was no significant difference in psoas MFI between any age group in males, MF-ES unit MFI was significantly higher in the 50–59 years group than any of the three younger groups (*p* < 0.01) and was also higher in the 40–49 years group compared with either of the two younger groups (20–29 years group: *p* < 0.05; 30–39 years group: *p* < 0.001).

There was no significant difference in vBMD between the 20–29 years and 30–39 years groups in either males or females. For the 40–49 years and 50–59 years groups, there was a statistically significant decline of vBMD with each advancing age-decade in both sexes (*p* < 0.01).

### Associations with bone mineral density and demographic variables

Correlations between age and psoas and MF-ES unit CSA were negligible or weak in both sexes and mostly not statistically significant (Table 3). While the same was true of psoas MFI, MF-ES unit MFI showed moderate and statistically significant correlations with age in both sexes (*p* < 0.001). Other demographic variables (height, weight, BMI, waist and hip circumference) correlated moderately or strongly with CSA measurements in both sexes (*p* < 0.001). In contrast, correlations with MFI measurements were weak or negligible in both sexes. L2–4 vBMD measurements were moderately correlated with age (*r* = −0.48 in both sexes) but had only weak or negligible correlations with other demographic variables. When compared with muscle measurements, vBMD correlated moderately with MF-ES unit MFI in both sexes (*p* < 0.001).

**Table 3.** Spearman rank correlation coefficients between paraspinal muscle CSA and MFI and spine vBMD with demographic characteristics by sex

	CSA				MFI				L2-4 vBMD		
	Female		Male		Female		Male		Female	Male	
	Psoas	MF-ES	Psoas	MF-ES	Psoas	MF-ES	Psoas	MF-ES			
**Age**	−0.10	0.03	−0.08	−0.03	0.17*	0.50^a^	0.13*	0.40^a^	−0.48^a^	−0.48^a^	
**Height**	0.20**	0.31^a^	0.15*	0.29^a^	0.11	0.02	−0.13*	−0.06	−0.10	−0.01	
**Weight**	0.32^a^	0.55^a^	0.35^a^	0.59^a^	0.12*	0.15*	0.04	0.16*	−0.15*	−0.09	
**BMI**	0.26^a^	0.41^a^	0.31^a^	0.50^a^	0.06	0.14*	0.09	0.17*	−0.10	−0.10	
**Waist circ**.	0.22^a^	0.45^a^	0.24^a^	0.45^a^	0.12*	0.25^a^	0.07	0.24^a^	−0.24^a^	−0.21**	
**Hip circ**.	0.22^a^	0.41^a^	0.26^a^	0.52^a^	0.04	0.22^a^	−0.04	0.11	−0.20*	−0.09	
**L2–4 vBMD**	0.04	−0.05	0.04	0.08	0.02	−0.38^a^	0.00	−0.32^a^	-	-	

CSA, cross sectional area; MF-ES, Multifidus and erector spinae as a unit; MFI, muscle fat index; vBMD, volumetric bone mineral density.

a *p*< 0.0001; **p < 0.001; *p < 0.05.

Following adjustment for MF-ES CSA, age, height, weight, waist and hip circumference, MF-ES MFI remained predictive of vBMD in females (β = −0.193, *p* < 0.001) and males (β = −0.157, *p* < 0.01). Following adjustment for the same variables, psoas MFI was not predictive of vBMD in females (β = 0.077, *p* = 0.116) or males (β = 0.053, *p* = 0.345). Following adjustment for MF-ES CSA alone, MF-ES MFI remained predictive of vBMD in females (β = −0.408, *p* < 0.001) and males (β = −0.338, *p* < 0.001). Psoas MFI was not predictive of vBMD after adjustment for psoas CSA in females (−0.021, *p* = 0.699) or males (β = 0.008, *p* = 0.892).

## Discussion

The major finding of this study was that fat infiltration of the MF-ES unit increased significantly with age in both sexes, and was associated with lower lumbar spine vBMD independent of demographic variables and CSA. Muscle CSA was not influenced by age at any region, and was not associated with lumbar spine vBMD. Our data suggest that muscle quality, and not muscle size, has an important role in supporting a favorable muscle–bone relationship.

Muscle fat infiltration is usually observed in association with age-related muscle atrophy,^26^ physical inactivity^27,28^ and chronic diseases such as diabetes and obesity.^29^ It is recognized as a predictor of declining strength and functional mobility,^30^ and the consequential loss of muscle quality is a component of sarcopenia.^31^ In the current study, we found significant increases in muscle fat infiltration of the MF-ES unit with age in both sexes, suggesting a progressive deterioration in muscle quality with age in healthy individuals. However, we did not observe corresponding muscle atrophy, suggesting that the process of intramuscular fat infiltration is an early architectural muscle change that precedes age-related loss of muscle mass and function. Our findings support the inclusion of muscle fat infiltration for investigations of sarcopenia, and as a key target for interventions aimed at improving muscle strength and functional performance with age.

Our study demonstrates that, independent of CSA, fatty infiltration of the paraspinal muscles increases with age in both sexes, and is associated with declining lumbar spine vBMD. It has long been recognized that muscle and bone comprise a functional unit, and that mechanical forces appear to dominate the integration and communication between the two organs.^32^ The reduced integrity of the paraspinal muscles resulting from increasing fat infiltration might lead to lower mechanical forces generated to the corresponding bone, and hence a suboptimum bone environment regardless of muscle size. There is also accumulating evidence that muscle acts as a secretory organ and that intramuscular fat is involved in inflammatory processes through secretion of proinflammatory cytokines^33^ which might negatively impact bone metabolism,^34^ although exact mechanisms are yet to be determined.

There were sex-specific differences in muscle fat infiltration. In females, paraspinal muscle degeneration characterized by greater intramuscular fat infiltration, appears to begin in the fourth decade of life, and in males the fifth decade, suggesting an earlier onset of muscle fat infiltration in females and a sex-dependent decline in muscle quality with age. It should also be noted that fat distribution in general differs substantially between the sexes and by age, with higher abdominal subcutaneous adipose tissue in females and higher visceral adipose tissue in males.^35^ The mechanisms underlying sex-specific differences in fat accumulation and ectopic fat distribution with aging are unclear. However, hormonal factors may play a role and excess accumulation of fatty acids around the muscle fibers may interfere with their functioning and reduce muscle quality.^36^

In the current study, lumbar paraspinal muscle CSA was greater in males than in females and did not decline with age. This is consistent with several studies that have shown age-related increases in the fat signal fraction in the erector spinae and multifidus muscles, but no age-related changes in CSA, in both sexes.^37,38^ This may reflect an age-related adaptation in muscle function and structure.^39^ The changes in muscle quality and size may also occur as a result of pathological degenerative processes, and not solely disuse atrophy associated with aging.^40^ The lack of association between paraspinal muscle CSA and lumbar spine vBMD contrasts with findings reported elsewhere of associations between muscle mass or CSA and vBMD.^41–43^ These studies mainly report associations between whole body muscle parameters and regional bone density, whereas in the current study we report localized associations specific to the spine. Our findings suggest that muscle fat infiltration, not muscle size or mass, is more closely related to bone density at the localized skeletal site.

This study also provides Chinese adult reference data for lumbar paraspinal muscle CSA and MFI in both sexes over the age range 20–59 years. Our values differ from those published for Caucasian and Japanese populations.^44–46^ In a Southern Chinese population, Crawford et al reported fatty infiltration values derived from *T*_1_ weighted MR images of the MF-ES unit of 31.5 (5.9)% in females [age 53.6 (6.9) years] and 26.3 (5.4)% in males [age 51.3 (8.1) years].^47^ These values for the MF-ES unit are higher than those reported here, where mean fat infiltration was 24.0 (9.3)% in males and 28.0 (7.3)% in females using a similar method of quantification. The differences may reflect heterogeneity in age between studies, but also the different techniques used to assess muscle mass and composition. For example, Fortin et al^22^ and Shahidi et al^38^ calculated fat fraction based on a single voxel placed in the center of the muscle, while our measurements included the entire muscle region. Differences in definition of the muscular ROI can also influence the outcome, since the CSA and MFI values are based on cross-sectional ROI’s with potentially different muscular border definitions. Our method of measuring the erector spinae and multifidus muscles as a single unit enables more time-efficient data collection by using imaging at L3–L4 to generalize for total lumbar paravertebral muscle fat content.

In the current study, we established both intra- and inter-rater reliability for the quantification of muscle CSA and MFI with excellent reproducibility similar to studies using *T*_1_ weighted MRI.^25,48,49^ Given that *T*_2_ weighted MRI images are frequently obtained in clinical examinations, the approach used in this study may be a clinically and economically viable method for assessing muscle quality in the lumbar spine.

This study has several limitations. Firstly, the cross-sectional design does not enable exploration of how changes in muscle quality and size might affect spine vBMD. Second, we did not investigate the influence of physical activity, sex hormone levels or years since menopause in the older age group, which are factors that may influence muscle fat infiltration.^27^ Third, our cohort comprised of adults aged 20–59 years and therefore data are not generalizable to older adults.

In conclusion, paraspinal muscle fat infiltration but not muscle CSA, increases with age in both sexes, and is related to lower lumbar spine vBMD in adults aged 20–59 years. This is the first study to demonstrate that independent of CSA and demographic variables, fat infiltration of the paraspinal MF-ES muscles is predictive of lower lumbar spine vBMD. We recommend that future studies exploring age-related changes in muscle and trajectory of bone loss include measurement of local muscle fat infiltration.
